# A Study of How Moral Courage and Moral Sensitivity Correlate with Safe Care in Special Care Nursing

**DOI:** 10.1155/2022/9097995

**Published:** 2022-07-13

**Authors:** Fateme Mohammadi, Banafsheh Tehranineshat, Afsaneh Ghasemi, Mostafa Bijani

**Affiliations:** ^1^Chronic Diseases (Home Care) Research Center and Autism Spectrum Disorders Research Center, Department of Nursing, Hamadan University of Medical Sciences, Hamadan, Iran; ^2^Community Based Psychiatric Care Research Center, Department of Nursing, School of Nursing and Midwifery, Shiraz University of Medical Sciences, Shiraz, Iran; ^3^Department of General Health, Fasa University of Medical Sciences, Fasa, Iran; ^4^Department of Medical Surgical Nursing, Fasa University of Medical Sciences, Fasa, Iran

## Abstract

**Background:**

Nursing is a caring profession, and nurses who have moral sensitivity and moral courage presumably can provide safe and better care for patients. This study aims at investigating how moral courage and moral sensitivity correlate with safe care in special care nursing.

**Methods:**

This study is a descriptive work of research. The participants consisted of 524 nurses who were in practice in the ICU (intensive care unit), CCU (C\coronary care unit), post-CCU (postcoronary care unit), and dialysis of four hospitals located in the south of Iran selected via census sampling. Data were collected from April to September 2020 using the moral sensitivity questionnaire (MSQ), professional moral courage questionnaire (PMCQ), and the assessment of safe nursing care questionnaire (ASNCQ). The collected data were analyzed using descriptive statistics, *t*-test, chi-square, multiple regression analysis, and Pearson's correlation coefficient in SPSS v. 22.

**Results:**

The mean ± SD of the nurses' age was 33.89 ± 6.91 years, and the mean ± SD of their work experience was 9.16 ± 4.67 years. The total mean score ± SD of the nurses' moral sensitivity was found to be 93.41 ± 2.68, the total mean score ± SD of their moral courage was found to be 96.38 ± 3.63, and the total mean score ± SD of their safe care scores was found to be 321.80 ± 9.76. The values of Pearson's correlation coefficients showed significant correlations between moral courage and safe care (*r* = 0.54, *p* < 0.001), moral sensitivity and safe care (*r* = 0.59, *p* < 0.001), and moral sensitivity and moral courage (*r* = 0.52, *p* < 0.001).

**Conclusion:**

There is a positive correlation between moral sensitivity and moral courage. Both positively correlated with special care nursing. Accordingly, through effective planning, education, and giving their support, nurse administrators can promote the abovementioned ethical virtues in the nursing staff, thereby improving the quality of care.

## 1. Introduction

Nurses are among the most important members of healthcare teams whose beliefs and attitudes have a significant impact on the performance of healthcare teams and the quality of care provided by them [1]. Caring is the essence of nursing and the primary and exclusive purpose of the profession [2, 3]. Nursing is defined as a caring profession with six main principles: compassion, trust, commitment, competence, communication, and courage [4, 5]. One of the factors that play a major role in the provision of safe, systematic, and quality care is moral courage [6]. Caring for critically or terminally ill patients, performing invasive care procedures, having to cope with emerging deadly diseases, etc. make it necessary for care providers, including nurses, to possess moral courage [7]. Moral courage means taking action in accordance with ethical values in spite of hardships and hazards in the face of moral challenges and dilemmas. An individual with moral courage consciously and voluntarily makes decisions and takes actions, which are beneficial to others despite all the negative consequences for them [8, 9].

Moral courage is a virtue that is essential for working conscientiously by all care providers, especially nurses. The increasing awareness of care receivers, changes in the healthcare needs of societies, social justice, and access to healthcare services have increased nurses' ethical distress, thus the need for these care providers to possess high levels of moral courage [10]. Due to the nature of their profession, nurses need to have the moral courage to provide safe, quality care, and avoid unethical behaviors [11]. Moral courage enables care providers to observe the ethical values and principles of their profession in such situations as maintaining patient privacy, breaking bad news, and caring for patients with infectious diseases [12, 13].

To provide safe care, in addition to possessing moral courage, care providers require awareness of ethical principles and moral sensitivity [14]. As one of the main components of professional competence in nursing, moral sensitivity helps care providers have patience, peace, and a sense of responsibility [15]. Moral sensitivity is the foundation of ethics in nursing and paves the ground for providing effective and ethical care to patients [16]. Care providers who have moral sensitivity care for their patients with devotion, they are sensitive to the physical and psychological needs of their patients, and they actively try to find ways of meeting those needs in a safe manner [17]. According to Khodaveisi et al., there is a significant positive relationship between moral courage and moral sensitivity and between moral courage and safe nursing care in nurses caring for COVID-19 patients. In addition, there is a significant positive relationship between moral sensitivity and safe nursing care [18].

One of the most important groups of nurses who provide care in critical or emergency situations where patients' lives are at serious risk is special care nurses [19]. Special care units sometimes have to provide care to patients whose conditions are critical. In this context, moral courage and moral sensitivity can ensure ethical decisions and safe care. Accordingly, this study investigates the correlation between moral courage and moral sensitivity on the one hand and safe care on the other in special care nursing.

## 2. Methods

### 2.1. Study Design and Setting

This study is a descriptive, cross-sectional work. The participants consisted of all the nurses who were in practice in the special care units (ICU, CCU, post-CCU, and dialysis) of four university hospitals located in the south of the province of Fars, Iran.

### 2.2. Population and Sample

Sampling lasted from April to September 2020 and was carried out according to the census sampling. Accordingly, 635 nurses in practice in special care units were invited to participate in the study; 524 of the participants completed and returned the questionnaires. Thus, the response rate was 82.51%. The inclusion criteria were being willing to participate and having at least one year's work experience. The subjects who failed to answer over half of the questions on the questionnaires and did not return the questionnaires were excluded.

### 2.3. Ethical Considerations

This study has been approved by the ethics committee of the Fasa University of Medical Sciences with the ethical code (IR.FUMS.REC.1399.098). All participants were informed of the objectives of the study and asked to sign the consent form. They were also assured that all their information would remain anonymous and confidential.

### 2.4. Data Collection

Data were collected using the following tools.

### 2.5. The Moral Sensitivity Questionnaire (MSQ)

Developed by Lutzen et al. in 1994, the moral sensitivity questionnaire consists of 25 items in 6 domains. Each item is scored on a 5-point Likert scale, ranging from “completely agree” (4 points) to “completely disagree” (0 points); the total score range is between 0 and 100. The 6 subscales of the questionnaire are as follows: (1) respect for patients' autonomy, (2) familiarity with the principles of communicating with patients, (3) professional knowledge, (4) experience of moral conflicts, (5) use of ethical concepts in ethical decision-making, and (6) honesty and benevolence [20]. A score between 0 and 50 indicates low moral sensitivity, a score between 51 and 75 indicates average moral sensitivity, and a score between 76 and 100 indicates high moral sensitivity. In Iran, the moral sensitivity questionnaire has been found to possess content and face validity. The reliability of the tool equals Cronbach's alpha of 0.83 [21].

### 2.6. The Professional Moral Courage Questionnaire (PMCQ)

The professional moral courage questionnaire was developed by Sekerka et al. in 2009. The scale consists of 15 items addressing 5 dimensions: moral agency (tendency to perform ethical acts and being prepared and able to face and cope with ethical issues) (3 items), multiple values (ability to combine one's own values with professional and organizational values and giving priority to professional values) (3 items), the endurance of threat (ability to identify threatening situations and to endure and overcome them) (3 items), going beyond compliance (willingness to perform ethical acts despite barriers and peripheral pressures at work) (3 items), and moral goals (setting personal goals based on respect, honesty, giving priority to patients, and accepted ethical values in the profession) (3 items). Each item is scored on a 7-point Likert scale, ranging from “never true” (1 point) to “always true” (7 points)—the total score range is between 15 and 105. A score between 15 and 50 indicates low professional moral courage, a score between 51 and 75 indicates average professional moral courage, and a score between 76 and 105 indicates high professional moral courage [22]. In Iran, Mahdaviseresht et al. verified the reliability of the scale with Cronbach's alpha of 0.81 [23].

### 2.7. The Assessment of Safe Nursing Care Questionnaire (ASNCQ)

Developed by Rashvand et al. in 2017, the assessment of safe nursing care questionnaire is a local instrument based on the healthcare system of Iran. The validity of the scale has been tested and verified by the faculty members of several medical schools. Cronbach's alpha of ASNCQ has been found to be 0.97, which demonstrates the high reliability of the scale. According to Rashvand et al., ASNCQ is a general scale that can be used for all subjects, free of inclusion criteria. The instrument consists of 32 items in 4 parts: part 1 addresses nursing skills (16 items), part 2 deals with the assessment of patients' psychological safety (4 items), part 3 deals with the assessment of patients' physical safety (7 items), and part 4 evaluates nurses' teamwork skills (5 items). All the items are scored on a 5-point Likert scale: never (1 point), seldom (2 points), sometimes (3 points), frequently (4 points), and always (5 points). The questionnaire also shows the loading of each item: the loading of items 14, 18, 19, 20, and 32 equals 1, the loading of items 2, 3, 4, 5, 7, 10, 11, 12, 13, 15, 16, 17, 21, 26, and 30 equals 2, the loading of items 1, 6, 8, 9, 23, 24, 25, 27, 29, and 31 equals 3, and the loading of items 22 and 28 equals 4. Thus, the respondent's score for each item is multiplied by the loading of that item and the resultant value is considered for evaluation. A score between 73 and 170 indicates poor performance, a score between 171 and 267 indicates average performance, and a score between 268 and 365 indicates satisfactory performance [24].

### 2.8. Data Analysis

The collected data were analyzed using descriptive statistics (frequency, percentage, mean, and standard deviation) in SPSS v. 22. The researchers used the chi-square test, independent *t*-test, and Pearson's correlation coefficients to study the relationship between moral courage, moral sensitivity, safe care, and demographic variables in special care nursing. The level of significance was set at *P* < 0.05. Next, the variables of demographics, moral courage, and moral sensitivity, which were found to correlate with safe care (*P* < 0.05), were entered into multivariate linear regression with the backward technique.

## 3. Results

A total of 524 nurses participated in this study. The mean ± SD of the nurses' age was 33.89 ± 6.91 years, and the mean ± SD of their work experience was 9.16 ± 4.67 years.  Table 1 shows the participants' demographic characteristics. The total mean score ± SD of the nurses' moral sensitivity was found to be 93.41 ± 2.68, the total mean score ± SD of their moral courage was found to be 96.38 ± 3.63, and the total mean score ± SD of their safe care scores was found to be 321.80 ± 9.76.  Table 2 shows the participants' mean scores for each dimension of moral sensitivity and moral courage. The calculated Pearson's correlation coefficients showed significant and direct correlations between safe care on the one hand and the demographic variables of age (*r* = 0.45, *p* < 0.001), and work experience (*r* = 0.42, *p* < 0.001).

Moreover, the Pearson's correlation coefficients showed a significant and direct correlation between the participants' moral courage scores and safe care scores (*r* = 0.54, *p* < 0.001). There were also significant and direct correlations between the participants' moral sensitivity and safe care (*r* = 0.59, *p* < 0.001) and moral sensitivity and moral courage (*r* = 0.52, *p* < 0.001).

The results of the study showed that among the domains of moral sensitivity, use of ethical concepts and familiarity with interpersonal communication skills had a small and negative correlation with safe care (*r* = −0.17, *p* < 0.008 and *r* = −0.27, *p* < 0.001, respectively). Among the domains of moral courage, moral goals and going beyond compliance had a negative and small correlations correlate with safe care (*r* = -0.24, *p* < 0.002 and *r* = −0.27, *p* < 0.001, respectively).

The results of multiple regression analysis showed that, overall, the variables of demographic characteristics, moral sensitivity, and moral courage explained 60% of the variance of the total safe care scores (Table 3).

## 4. Discussion

This study was conducted to investigate the correlation between moral sensitivity and moral courage on the one hand and safe nursing care on the other. The results of the study showed that moral sensitivity and moral courage significantly correlated with safe care. In addition, there was a significant correlation between moral sensitivity and moral courage.

In the study of Kleemola et al., the nurses state that the situations in which moral courage is required are centered on safe care. Such situations are often related to having a verbal communication, engaging with patients, and engaging with managers and doctors. The participants also state that moral courage-based care is characterized by showing respect for patients, alleviating their pain, treating patients equally, and maintaining patients' dignity [25]. According to Gallagher, promotion of moral courage can improve the quality of healthcare [26]. Nurses who possess moral courage are always present at their patients' side, view their patients as human beings with various needs, empathize with their patients, and care about their interactions with their patients [27, 28]. Thus, by showing moral courage when defending patients' rights, nurses can have a direct impact on patients' safety [29].

The findings of the present study showed the moral courage status of the nurses to be satisfactory. The participants' highest mean scores were for the following dimensions of moral courage (in descending order): moral goals, tolerating the threat, and moral agency. Only the dimension of moral goals was found to significantly correlate with safe care. The dimension of moral goals is associated with the use of goal-setting strategies toward benefiting others, giving priority to others' needs over one's own needs, and following ethical values and principles in practice [30].

Endurance of threat is related to the ability to cope with the reactions of one's colleagues and the significance of maintaining one's professional and personal status [31]. Moral agency is connected with having a sense of responsibility and managing difficult situations. This appears to be an important dimension as nurses with greater moral agency provide better care to patients because they are better capable of managing and resolving moral conflicts in complex ethical situations [32]. It seems that the application of goal-setting strategies by the special care nurses in the present study has been more significant in their observance of ethical principles toward providing safe care.

Another finding of the present study is a significant correlation between moral sensitivity and safe care: the higher the nurses' moral sensitivity, the safer the nature of care they give. Similarly, Mohammadi et al. reported that nurses who possess enough moral sensitivity could create an atmosphere in which patients not only have their rights respected but also feel safe in these conditions, and the goals of healthcare can be accomplished [33]. Amiri et al. do not report a significant correlation between moral sensitivity and safe care, but they report a significant inverse correlation between “experience of ethical conflicts”—one of the domains of moral sensitivity—and quality of care. It appears that nurses who practice ethical decision-making are faced with a conflict between their personal and ethical values and, therefore, suffer from ethical tension. If this tension is not coped with properly, it can lead to nurses' distancing themselves from their patients and feeling indifferent to ethical care [34]. Stressing the significance of moral sensitivity in their study, Razzani et al. state that in order for ethical principles and rules to be observed in healthcare environments, caregivers need to be aware of and sensitive to these principles—only then can care be provided effectively [35]. According to the study of Escolar-Chua, there is a significant positive correlation between nursing students' moral sensitivity and ethical tension: the students who had experienced ethically distressing situations had an understanding of other caregivers' performance and the ethical factors that affected patients' conditions. In conclusion, the nursing profession is inherently sensitive to other people's needs [36].

The findings of the present study showed the moral sensitivity mean score of the nurses to be satisfactory. Among the domains of moral sensitivity, honesty and benevolence, use of ethical concepts, and familiarity with interpersonal communication skills were the areas where the participants scored the highest. The results also showed that the dimensions of use of ethical concepts and familiarity with interpersonal communication skills significantly correlated with safe care. In most studies conducted in other countries, the nurses' highest mean scores are for the use of ethical concepts [37, 38]. In the study of McDonald et al., the interactions between nurses and patients are often influenced by the dominant work culture that is the outcome of the relationships between nurses, their colleagues, the authorities, patients, and the organization [39]. According to a study in Iran, compliance with ethical principles in clinical decision-making is not satisfactory and nurses are not capable of employing their ethical knowledge in real settings [40]. In the present study, however, the moral sensitivity scores of the nurses were satisfactory and honesty and benevolence were considered to be important. The culture of supporting nurses in the special care units in the present study caused the nurses to promote ethical practice and interpersonal communication. According to the study of Amiri et al., the moral sensitivity mean score of nurses in internal wards is considerably high [41]. The findings of the study of Ohnishi et al. show that nurses in the psychiatrist units of hospitals in Finland and Japan have high levels of moral sensitivity [42].

On the other hand, a few studies report nurses' moral sensitivity to be average or below average [40–43]. This discrepancy can be attributed to cultural and organizational differences and, more importantly, to differences between diseases and care units. Patients' physical and psychological conditions can influence nurses' moral sensitivity—the nurses who are in practice in special care and psychiatric units report higher levels of moral sensitivity than the nurses in other units [44]. The findings of the present study also show a statistically significant correlation between moral courage and moral sensitivity. Likewise, Watkins et al. report a significant positive correlation between moral courage and moral sensitivity [45]. This means that an increase in nurses' moral sensitivity correlates with an increase in their moral courage. In the study of Hannah et al., moral courage is reported to significantly correlate with ethical behaviors [46].

In addition, it seems that moral sensitivity can cause ethical distress and if nurses possess moral courage, acting ethically will reduce their ethical distress. All these factors are indirectly inter-related [47, 48].

The findings of the present study show a positive relationship between special care nurses' work experience and age on the one hand and moral courage and moral sensitivity on the other, which is consistent with the study of Ebadi et al. This relationship can be attributed to more experienced nurses' better familiarity with organizational conditions, higher professional and moral competence, and adoption of courageous behaviors displayed by their colleagues in clinical environments [49]. Similarly, the results of the study by Khodavesi et al. show that age and work experience positively correlate with moral sensitivity, moral courage, and safe nursing care in COVID-19-specific hospital departments. However, Khodavesi et al. did not find the relationship between age on the one hand and moral sensitivity and moral courage on the other to be significant, which is inconsistent with the findings of the present study [18].

Hanifi et al. report a significant relationship between moral courage and gender in nursing students [50]. Similarly, a few other studies claim that gender influences individuals' level of awareness regarding ethics and current norms: men are more likely to display courageous behaviors, while women tend to behave more humbly and conservatively [23–51].

According to the findings of the present study, safe care significantly correlates with age, gender, education, work experience, and the unit where nurses work. A safe healthcare system relies on measures that ensure nursing interventions are free from error and any unwanted consequences. In the study of Kim, the results show a significant relationship between nurses' education, age, professional experience, experience of safety accidents, and job satisfaction on the one hand and safe care activities on the other [52].

Similarly, Mwachofi et al. reported a significant correlation between nurses' perception of safe care and their age: older nurses are less familiar with safe care [53]. Kalantari et al. did not find the relationship between nurses' place of work, gender, type of employment, work shifts, and participation in workshops on the one hand and their professional performance on the other to be statistically significant [54]. However, the study of Beeman titled “The Educational Needs and Management of Military Nurses,” shows a significant correlation between nurses' providing safe care and their age and work experience [55]. The majority of the nurses who participated in the present study were young and, since education on codes of ethics in patient care has been added to the nursing curriculum in recent years, the young and less experienced nurses seemed to have a better understanding of moral sensitivity and moral courage in providing care to patients. In addition, nurses with higher education have a better understanding of moral sensitivity and moral courage in providing safe care.

The results of the study showed that the most powerful predictor of safe care was the unit where the nurses worked. The second best predictor of safe care was nursing skills. Special care units are among the most sensitive hospital environments where nurses play a significant role. However, the available literature does not provide much information about clinical measures intended to increase the safety of patients in special care units [56]. The study of Jin and Yi recommends that healthcare personnel should be given continuous education and that their critical thinking and teamwork skills need to be improved in order for safe care to happen [57]. According to another study, life-threatening situations and lack of quick access to a doctor can increase the rate of error if nurses' knowledge and skills are inadequate [58]. Thus, the development of nurses' knowledge and skills will enhance patients' safety and the quality of care provided to patients in special care units. The novelty of the subject of the present study made it difficult to discuss the findings of the study in comparison with other studies.

### 4.1. Limitations

One of the limitations of the present study is that the questionnaires were completed on a self-report basis and the respondents' answers to the items may not have been accurate. In addition, the present study exclusively addressed nurses. It is suggested that future studies evaluate other members of healthcare teams, including doctors. It is also suggested that a similar study be carried out in other countries.

### 4.2. Implications for Clinical Practice

Moral courage contributes to nurses' moral sensitivity in their clinical practice. As nurses who possess high levels of moral courage and sensitivity take better care of patients, nursing managers are recommended to take steps to educate nurses about ethical codes and moral sensitivity and help them develop moral courage so that they can provide safe nursing care.

## 5. Conclusion

There is a positive correlation between moral sensitivity and moral courage. Both are positively correlated with special care nursing. Accordingly, through effective planning, education, and giving their support, nursing managers are recommended to pay special attention to moral sensitivity and moral courage as qualities that can improve the quality of care and ensure safe care.

## Figures and Tables

**Table 1:** The demographic characteristics, moral sensitivity' scores, moral courage' scores, and safe care' scores based on the demographic characteristics.

Variable		N (%)	Moral sensitivity Mean ± SD	Moral courage Mean ± SD	Safe care Mean ± SD
Gender	Male	209 (39.9)	80.24 ± 2.11	91.25 ± 2.87	316.31 ± **9**.24
	Female	315 (60.1)	82.28 ± 2.08	90.38 ± 2.44	317.51 ± 9.11

Marital status	Single	133 (25.4)	88.21 ± 2.35	92.55 ± 3.07	318.28 ± 9.08
	Married	350 (66.8)	83.47 ± 2.13	90.02 ± 2.97	315.32 ± 9.47
	Widowed	24 (4.6)	81.24 ± 2.20	89.25 ± 2.13	311.43 ± 9.61
	Divorced	17 (3.2)	80.32 ± 2.31	87.32 ± 2.87	310.55 ± 9.28

Education	Bachelor's degree	507 (96.8)	87.17 ± 2.24	94.15 ± 3.11	318.61 ± 8.97
	Master's degree	17 (3.2)	82.44 ± 2.17	90.05 ± 2.71	315.24 ± **9.10**

Place of work	ICU	139 (26.5)	94.37 ± 2.42	96.15 ± 2.54	317.84 ± 9.32
	CCU	131 (25.3)	93.11 ± 2.13	95.43 ± 2.17	315.77 ± 9.46
	Post-CCU	138 (26.1)	93.47 ± 2.54	93.25 ± 2.07	313.44 ± 9.07
	Dialysis	116 (22.1)	89.32 ± 2.43	90.27 ± 2.44	313.01 ± 9.28

Age (years)	23–33	289 (55.2)	83.47 ± 2.13	93.05 ± 2.74	317.71 ± 9.02
	34–44	183 (34.9)	87.24 ± 2.57	91.08 ± 2.63	314.32 ± 9.27
	45–55	52 (9.9)	81.75 ± 2.56	87.11 ± 2.57	311.65 ± 9.13

Work experience (years)	1–5	119 (22.7)	83.24 ± 2.33	90.14 ± 2.37	313.81 ± 9.01
	6–10	223 (42.6)	87.39 ± 2.28	94.25 ± 2.45	317.64 ± 9.39
	11–15	129 (24.6)	85.07 ± 2.25	90.07 ± 2.66	312.24 ± 9.31
	More than 16	53 (10.1)	80.17 ± 2.21	87.64 ± 2.33	311.75 ± 9.61

**Table 2:** Means and standard deviations of the participants' scores for the domains of moral sensitivity, moral courage, and safe care.

variable		Minimum	Maximum	Mean	SD
Moral sensitivity	Respect for patient autonomy	9.00	12.00	10.99	1.07
	Familiarity with communication skills	16.00	20.00	19.32	0.81
	Professional knowledge	5.00	8.00	7.24	0.85
	Experience of ethical conflicts	9.00	12.00	10.98	0.64
	Use of ethical concepts	15.00	20.00	18.64	1.06
	Honesty and benevolence	23.00	28.00	26.21	1.10

Moral courage	Moral agency	14.00	21.00	18.60	1.89
	Multiple values	12.00	21.00	18.39	2.44
	Endurance of threat	17.00	21.00	20.14	0.73
	Going beyond compliance	14.00	21.00	19.30	1.63
	Moral goals	17.00	25.00	21.35	1.71

Safe care	Assessment of nursing skills	141.00	168.00	154.77	6.47
	Evaluation of psychological safety	18.00	25.00	22.56	1.88
	Evaluation of physical safety	75.00	97.00	88.62	5.75
	Evaluation of teamwork skills	41.00	63.00	55.83	4.91

**Table 3:** Predictors of safe care in special care nursing.

variable	Unstandardized coefficients		Standardized coefficients	*t*	Confidence intervals	*P*-value
	B	Std. Error	Beta			
Moral sensitivity	0.59	0.13	0.57	4.38	0.35, 1.04	<0.001
Moral courage	0.54	0.18	0.44	2.44	0.21, 0.96	<0.001
Gender	0.40	0.15	0.28	1.86	0.16, 0.91	0.007
Age	0.04	0.11	0.16	1.45	0.12, 0.82	0.013
Marital status	0.12	0.11	0.14	1.27	0.12, 0.83	0.285
Education	-0.39	0.19	0.24	1.26	0.20, 0.87	0.197
Work experience	-0.13	0.25	0.36	1.44	0.21, 0.82	0.003

## Data Availability

The data supporting the findings of the present study are available from corresponding author upon request.
